# Development of Irinotecan Liposome Armed with Dual-Target Anti-Epidermal Growth Factor Receptor and Anti-Fibroblast Activation Protein-Specific Antibody for Pancreatic Cancer Treatment

**DOI:** 10.3390/pharmaceutics14061202

**Published:** 2022-06-05

**Authors:** Hung-Jun Lin, Tien-Li Liang, Yao-Yuan Chang, Der-Zen Liu, Jia-Yu Fan, Steve R. Roffler, Shyr-Yi Lin

**Affiliations:** 1 Graduate Institute of Biomedical Materials and Tissue Engineering, College of Biomedical Engineering, Taipei Medical University, Taipei 110, Taiwan; d02548004@g.ntu.edu.tw (H.-J.L.); m225100009@tmu.edu.tw (Y.-Y.C.); tonyliu@tmu.edu.tw (D.-Z.L.); hjlindaniel@gmail.com (J.-Y.F.); 2 Department of Nursing, Hungkuang University, Taichung 433, Taiwan; b210096025@tmu.edu.tw; 3 Medical and Pharmaceutical Industry Technology and Development Center, New Taipei 248, Taiwan; 4 Institute of Biomedical Sciences, Academia Sinica, Taipei 115, Taiwan; dannym0814@hotmail.com; 5 Department of General Medicine, School of Medicine, College of Medicine, Taipei Medical University, Taipei 110, Taiwan; 6 Division of Gastroenterology, Department of Internal Medicine, Wan Fang Hospital, Taipei Medical University, Taipei 110, Taiwan; 7 TMU Research Center of Cancer Translational Medicine, Taipei Medical University, Taipei 110, Taiwan

**Keywords:** pancreatic cancer, liposomal drug, targeted liposome, bispecific targeting antibody

## Abstract

Pancreatic cancer is one of the most common causes of death in Taiwan. Previous studies have shown that more than 90% of pancreatic cancer cells presented epidermal growth factor receptor (EGFR) cell marker, and this marker is thought to be important as it is related to activation of cancer cell proliferation, angiogenesis, and cancer progression. Moreover, tumor-associated fibroblasts were involved in tumor proliferation and progression. In this study, we fabricated an anti-EGFR and anti-fibroblast activation protein bispecific antibody-targeted liposomal irinotecan (BS−LipoIRI), which could specifically bind to pancreatic cancer cells and tumor-associated fibroblasts. The drug encapsulation efficiency of BS−LipoIRI was 80.95%, and the drug loading was 8.41%. We proved that both pancreatic cancer cells and fibroblasts could be targeted by BS−LipoIRI, which showed better cellular uptake efficacy compared to LipoIRI. Furthermore, an in vivo mouse tumor test indicated that BS−LipoIRI could inhibit pancreatic cancer growth up to 46.2% compared to phosphate-buffered saline control, suggesting that BS−LipoIRI could be useful in clinical cancer treatment.

## 1. Introduction

Pancreatic cancer is one of the leading causes of cancer-related death in Taiwan, and approximately 300,000 people die from pancreatic cancer annually worldwide [1]. The diagnosis of pancreatic cancer is challenging because effective screening methods and obvious symptoms are lacking. The current clinical treatment is mainly surgical resection, supplemented by chemotherapy and radiotherapy [2]. Unfortunately, the overall 5-year survival rate of pancreatic cancer has been the lowest among all cancers for a long time. The 5-year survival rate only increased from 4% in 1987 to 9% in 2012, showing the difficulty of treatment [3]. Therefore, developing more effective treatment methods is a critical problem in clinical practice.

Pancreatic cancer usually proliferates asymptomatically in the body with invasion and spread; thus, it is difficult to detect in the early stage [4]. Therefore, it is urgent to develop a more specific targeting drug for tumor reduction than that in other cancers. The surface of pancreatic cancer cells often has special manifestations. Epidermal growth factor receptors (EGFRs) are often found on the surface of 90% of pancreatic cancer cells and induce cell proliferation, migration, and angiogenesis with overexpression [5]. Neutralizing monoclonal antibodies, such as cetuximab, and small molecule tyrosine inhibitors are used to inhibit EGFR function in cancer treatment. Previous randomized clinical trials have shown that addition of EGFR-targeting drugs could significantly improve the marginal benefit for cancer treatment [6,7]. However, cancer-associated fibroblasts are usually activated with pancreatic cancer proliferation. The cancer-associated fibroblasts accelerate the proliferation, metastasis, and invasion of cancer cells and are activated by surface fibroblast activation protein (FAP) expression and TGF-β cytokine and vascular epithelial growth factor secretion [8,9]. Moreover, they could generate abnormal collagen to form physical barriers to prevent penetration of chemotherapeutic drugs into tumor cells. Therefore, it is important to develop a dual-targeting agent for both pancreatic cancer cells and cancer-associated fibroblasts.

Irinotecan is a derivative of camptothecin, which is extracted from the Chinese tree camptotheca acuminate [10]. IRI is a water-soluble drug, which inhibits the resealing of single-strand DNA breaks mediated by topoisomerase I by stabilizing cleavable complexes [11,12]. IRI has proven antitumor ability for colorectal cancer and pancreatic cancer treatment. However, the dose-limiting toxicities are known to be leukopenia and diarrhea. It is also believed that cardiovascular toxicity occurs very rarely [13,14]. A higher risk of toxic effects is present when the body has higher circulating free drug concentrations.

The liposome is an effective cancer drug carrier for cancer treatment. The advantages of great biocompatibility, prolonged drug effective time, and penetration of tumor area by the enhanced permeability and retention (EPR) effect are increasing the efficacy of cancer treatment [15]. Furthermore, the particle surface could modify specific targeting antibodies, which increases the effectiveness of anticancer drugs in killing cancer cells. Several agents can be used to modify liposomes so that they can actively target cancer cells, such as peptides, receptor ligands, antibodies, specific antibody fragments, and proteins [16,17]. However, the specific targets of short peptide chains are inadequate in the surface modification of liposomes. The modification of traditional antibody molecules on the particle surface would greatly increase their particle size, which reduces the efficiency of liposomes through the EPR effect. Moreover, the Fc fragment of the traditional antibody would easily attract immune cells to clear liposomal drugs [18]. Therefore, this study has completed the construction of dual-recognition specific antibodies (anti-EGFR antibody for pancreatic cancer cells and anti-FAP for fibroblasts surrounding pancreatic cancer cells), which remove the Fc fragment and preserve the specific target region, Fv fragments. Dual-targeting specific antibodies are genetically engineered to contain the anti-mPEG region, which recognize the mPEG molecule of liposomal drugs. This study aimed to provide a novel strategy for specific targeting for pancreatic cancer. We first developed dual-targeting (anti-EGFR and anti-FAP) irinotecan liposomes for both pancreatic cancer cells and tumor-associated fibroblasts. The anti-EGFR or anti-FAP bispecific antibody-targeted liposomal irinotecan (BS−LipoIRI) were examined for both in vitro and in vivo specific targeting ability and tumor growth inhibition effect.

## 2. Materials and Methods

### 2.1. Production of Dual Target Anti-EGFR/Anti-FAP Specific Antibody

The dual-targeting anti-EGFR/anti-FAP specific antibody was constructed through genetic engineering and provided by Kuo-Hsiang Chuang’s laboratory. Briefly, the genes of the light chain (anti-EGFR and anti-FAP) and heavy chain (anti-mPEG) were incorporated into a single plasmid. The plasmid vectors were transfected into the cells (Expi293 cells) for large production. After 5 days, the supernatant of Expi293 cells was harvested for protein purification by HisTrap columns. The antibody concentrations were evaluated by BCA (bicinchoninic acid assay).

### 2.2. Characterization of Irinotecan Liposomes

Liposomal irinotecan (LipoIRI) was prepared by thin-film hydration method. The lipid thin films were obtained in the ethanol solutions with an 8.6:5.7:0.7 lipid molar ratio of 1,2-dioctadecanoyl-sn-glycero-3-phosphocholine (DSPC) to cholesterol to DSPE-mPEG-2000 through the rotary evaporation. The lipid thin films were hydrated by the addition of phosphate-buffered saline (PBS) containing 10 mg/mL of irinotecan, and multilamellar lipid vesicles were formed by vortex dispersion. Next, the lipid vesicle solution was extruded 10 times for each pore size (1000 nm, 800 nm, 600 nm, 400 nm, 200 nm, and 100 nm) through polycarbonate filters to form Lipo-IRI. Then, the LipoIRI was used as described in the following experiments, and each experiment was conducted in triplicate.

The particle size, PDI, and zeta potential were examined by BIC 90Plus Particle Size Analyzer following the manufacturer’s protocols. The drug loading and encapsulation of Lipo-IRI were measured by high-performance liquid chromatography (HPLC) system. Each sample was diluted to an appropriate concentration before injection into a C18 column (5 μm, 4.6 × 150 mm, GL Science) and then determined by the wavelength of 375 nm. The drug loading and drug encapsulated efficiency were calculated by the software using the following formula: DL (%) = W_a_/W_b_ × 100%; EE (%) = W_a_/W_c_ × 100% (W_a_, the drug amount encapsulated in liposomes; W_b_, the total lipid amount of liposomes; W_c_, the drug amount used in the liposomal preparation).

### 2.3. In Vitro Targeting Ability of Specific Antibody Liposomes

To analyze the anti-EGFR/anti-FAP antibody binding affinity on the surface of the liposome, anti-PEG antibody (AGP4) (5 μg/plate well) was coated in an enzyme-linked immunosorbent assay (ELISA) plate with coating buffer (0.1 M NaHCO_3_, pH 9) at 4 °C overnight. Then, the ELISA plates were blocked with skim milk (5%) at 37 °C for 4 h. The antibodies were diluted with skim milk (2%) serially to different concentrations, and then incubated with LipoIRI for 2 h (50 μL/plate well). After washing with PBS, we added 50 μL of mouse anti-His antibodies, HRP-conjugated goat anti-mouse IgG Fc and 2,2′-azino-di-(3-ethylbenzthiazoline sulfonic acid) substrate (ABTS) to the well. Then, we measured the wavelength at 405 nm using the ELISA reader.

BxPC3/EGFR^+^ cells and WS-1/FAP^+^ cells were cultured in the plates (3 × 10^5^ cells per well). After one day, the BxPC3 or WS-1 cells were treated with the liposomal drugs (50 μL, diluted serially with medium containing 0.05% bovine serum albumin) for 1 h at 37 °C. Then, using medium for washing, the plate wells were incubated with anti-PEG mAb (AGP4-biotin, 50 μL, 5 μg/mL), horseradish peroxidase-conjugated streptavidin (1 μg/mL), and ABTS substrate. The data were measured at 405 nm using the ELISA reader.

### 2.4. Drug Release and Cellular Uptake Studies

The in vitro drug release profile of irinotecan was examined, and the sample solution containing LipoIRI or BS−LipoIRI (2 mL, 10 mM of IRI) was added into the dialysis bag (MWCO 3500, Cellu-Sep^®^ T1, Orange Scientific, Seguin, TX, USA). The dialysis bag was then soaked in 300 mL of PBS buffer with 0.5% Tween 80 with shaking of 150 rpm at 37 °C. Moreover, 20 mL of PBS buffer was collected at 1, 2, 4, 6, 8, 10, 24, and 48 h and replaced with 20 mL of fresh PBS. The drug concentration of released irinotecan was examined by the HPLC system.

For the cellular uptake assay, BxPC3 was incubated with DiI-labeled LipoIRI or DiI-labeled BS−LipoIRI for 15 min, 30 min, 1 h, 3 h, 8 h, and 24 h. After that, the BxPC3 cells were collected and washed with PBS three times and then analyzed using flow cytometry. The results were presented as the mean fluorescent intensity (MFI) for the 10,000 collected cells.

To understand the mechanism of BS−LipoIRI cellular uptake by BxPC-3 cells, the cells were cultured for 2 h with methyl-b-cyclodextrin (MBCD) (0.5 mM, an inhibitor of caveolae-mediated endocytosis), amiloride (50 μM, an inhibitor of micropinocytosis), cytochalasin D (10 μg/mL, an inhibitor of micropinocytosis and phagocytosis), and sucrose (450 mM, an inhibitor of clathrin-mediated endocytosis) separately. After incubation, the cells were measured for the mean fluorescence intensity by flow cytometry.

### 2.5. In Vitro Pancreatic Tumor Cell Viability

BxPC3/EGFR cells were cultured in 96-well plates at 5 × 10^3^ cells per well. After one day, the cells were incubated with BS−LipoIRI (50 μL, diluted serially with a medium containing 0.05% BSA) for 60 min at 37 °C. After removal of the BS−LipoIRI, the BxPC3 cells were cultured in a growth medium for 72 h at 37 °C. The survival of cells was examined with an MTT assay. Absorbance measurements at 570 nm were used by the ELISA reader

### 2.6. In Vivo Drug Pharmacokinetic and Distribution Studies

All animal experiments were approved by the Institutional Animal Care and Use Committee (Approval No.: LAC-2017-0335). Eight-week-old female Sprague Dawley (SD) rats were administered one dose of 20 mg/kg of irinotecan, LipoIRI, or BS−LipoIRI via jugular vein injection. The blood samples were collected from the jugular vein of the rats in heparinized tubes at different time points (5 min, 15 min, 30 min, 1 h, 2 h, 4 h, 6 h, 8 h, 12 h, 24 h, and 72 h) after drugs administration. The samples were then centrifuged at 3000 rpm for 15 min by centrifuge to obtain plasma and analyzed by liquid chromatography-tandem mass spectrometry (LC-MS/MS). 

Bio-distribution studies of mice were examined in BxPC3/EGFR^+^: WS1/FAP^+^-bearing SCID mice. After the volume of tumor size reached 200 mm^3^, the mice were administered BS−LipoIRI. The mice were administered irinotecan (20 mg/kg) intravenously through the tail vein. After 8 h, the mice were sacrificed by CO_2_ asphyxiation. Next, the mice were transcardially perfused with PBS buffer containing heparin (10 IU) until the organs were cleared of blood. Then, we collected the major organs (heart, lungs, liver, kidneys, spleen, and tumors), which were harvested and weighed. The drugs were extracted from the organs, which were homogenized with PBS/10 IU heparin solution (500 μL). The irinotecan drug extractions were analyzed by LC-MS/MS.

### 2.7. Administration of BS–LipoIRI against Human Pancreatic Tumor

All mice were bred in a specific pathogen-free (SPF) animal facility. Eight-week-old SCID mice received BxPC3 cells and WS1 cells (1 × 10^6^ cells, 1:1) by subcutaneous injection. When the tumor volumes were approximately 150 mm^3^, the mice were divided into four groups randomly (saline, LipoIRI, BS−LipoIRI 1:100, BS−LipoIRI 1:200; 7 mice per group). The mice were treated with 20 mg/kg of irinotecan through the tail vein twice a week for 3 weeks. The tumor size was calculated using the following formula [19]:

Tumor volume (mm^3^) = length × (width^2^)/2

### 2.8. Statistical Analysis

All data were reported as mean and standard deviation (SD). The significance of the data was analyzed using Mann–Whitney U tests. Statistical analysis was performed using GraphPad Prism (GraphPad Software, San Diego, CA, USA), and a *p*-value < 0.01 was considered statistically significant.

## 3. Results

### 3.1. Characterization and Stability of LipoIRI

**Figure 1:**
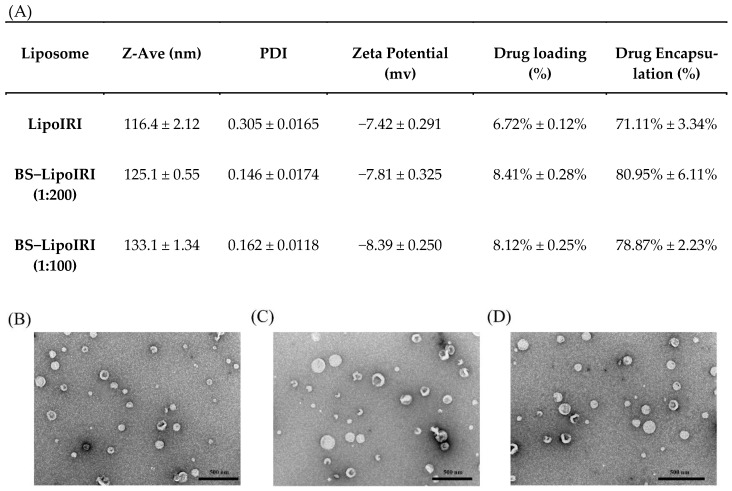
(**A**) Physicochemical characteristics of LipoIRI, BS−LipoIRI (1:200), and BS−LipoIRI (1:100). TEM images of (**B**) LipoIRI, (**C**) BS−LipoIRI (1:200), and (**D**) BS−LipoIRI (1:100). Results are expressed as mean ± SE of three independent experiments.

Liposomes were prepared using the thin-film hydration method, and the particle size was controlled by extraction. The data are shown in Figure 1. The average sizes of LipoIRI, BS−LipoIRI (1:200), and BS−LipoIRI (1:100) were 116.4 nm, 125.1 nm, and 133.1 nm, respectively. The encapsulation efficiency of the drug was 70–80%, and drug loading was 6%–8%. The polydispersity index (PDI) and zeta potential of LipoIRI, BS−LipoIRI (1:200), and BS−LipoIRI (1:100) were 0.305 and −7.42 mV, 0.146 and −7.81 mV, and 0.162 and −8.39 mV, respectively. The results revealed that the particle sizes are approximately 120 nm. No significant difference was observed in the mean particle diameter or encapsulation efficiency among the three formulations. The structure of LipoIRI or BS−LipoIRI were observed by TEM examination (Figure 1B–D), and all groups exhibited spherical morphology and were well dispersed and separated. 

To examine the in vitro stability of BS−LipoIRI (1:100), they were incubated in PBS to monitor for 6 weeks, and the average size was maintained at 115.7 nm (Table 1). However, the PDI of BS−LipoIRI was 0.592, indicating that the particle size of the solution did not show monodispersity. This result indicated that the best storage life of BS−LipoIRI (1:100) was 4 weeks. 

### 3.2. In Vitro Dual Targeting of BS−LipoIRI

**Figure 2:**
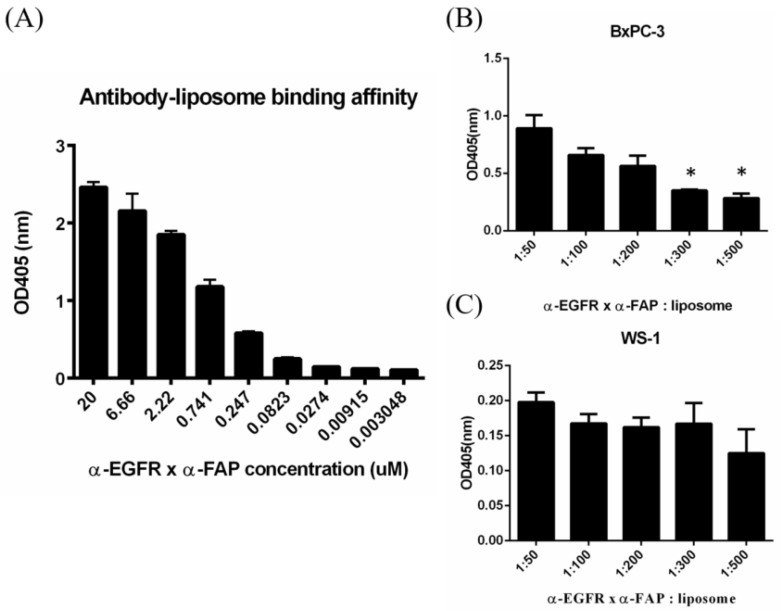
Assessment of binding affinity of BS−LipoIRI. (**A**) Different concentrations of anti-EGFR/anti-FAP antibody for LipoIRI. (**B**) BxPC3/EGFR cellular binding affinity and (**C**) WS1/FAP cellular binding affinity. (Data are presented as mean ± SD, *: *p* < 0.01.)

The anti-EGFR/anti-FAP antibody could be bound to the LipoIRI by anti-mPEG Fab fragment. To investigate the efficiency of antibody that is non-covalently bound to the LipoIRI, sandwich ELISA was performed. The anti-PEG backbone AGP4 mAbs, which could specifically bind to the backbone of the PEG chain, were coated in 96-well plates to capture LipoIRI with or without antibody modification. The HRP-conjugated secondary antibody was added to detect antibodies on LipoIRI, and HRP activity was determined by absorbance at 405 nm (OD value) due to the oxidation product of ABTS. The results demonstrate that the binding concentration of BS−LipoIRI in the formulations increased, leading to an increase in the resulting absorbance, indicating that antibodies were noncovalently bound to LipoIRI (Figure 2A). 

To determine the cellular binding affinity of BS−LipoIRI to BxPC3/EGFR and WS1/FAP, different molar ratios of anti-EGFR/anti-FAP antibody: LipoIRI were examined. In BxPC3 cells, the 1:300 and 1:500 groups had lower binding affinity than any other groups (Figure 2B). Conversely, BS−LipoIRI showed great cellular binding affinity in all groups (Figure 2C). Thus, we selected molar ratios of 1:200 and 1:100 BS−LipoIRI for further experiments.

### 3.3. In Vitro Drug Release Studies

**Figure 3:**
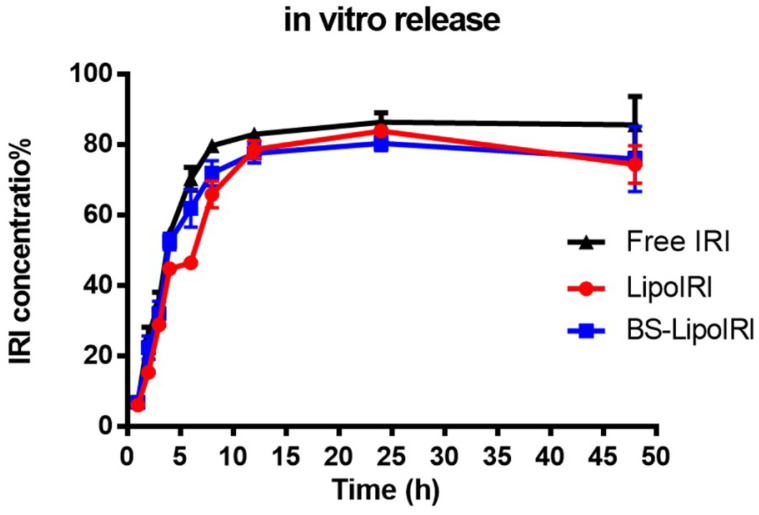
Drug release profile of irinotecan of LipoIRI and BS−LipoIRI.

The drug release profile of BS−LipoIRI and LipoIRI was evaluated at pH 7.4 PBS. As depicted in Figure 3, the cumulative release of irinotecan reached a plateau after 24 h. Within 24 h, the release rate of the BS−LipoIRI was similar to that of LipoIRI, which indicated that modification of the anti-EGFR/anti-FAP antibody did not affect the drug release. The results showed that there was no significance between Free IRI, LipoIRI, or BS−LipoIRI. 

### 3.4. Cellular Uptake and Mechanism of the BS−LipoIRI

**Figure 4:**
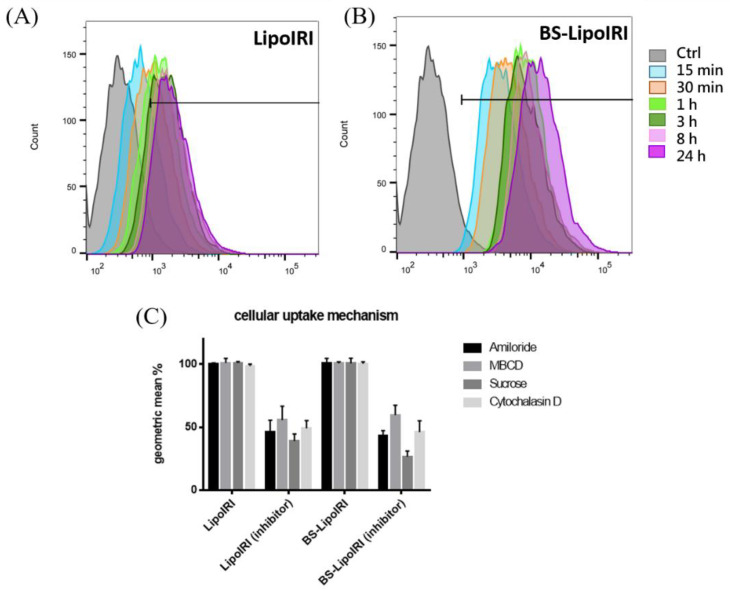
Cellular uptake of the (**A**) DiI-labeled LipoIRI or (**B**) DiI-labeled BS−LipoIRI was examined after incubation of cells at 15 min, 30 min, 1 h, 3 h, 8 h, and 24 h. (**C**) BxPC3 cells were treated with DiI-labeled LipoIRI or BS−LipoIRI for 2 h in the presence of various inhibitors or without inhibitors.

To examine the targeting ability of the BS−LipoIRI to BxPC3/EGFR pancreatic tumor cells, the cellular uptake of the DiI-labeled LipoIRI or DiI-labeled BS−LipoIRI was examined after incubation of cells at different time intervals. As shown in Figure 4, the cellular uptake amount of the BS−LipoIRI at all-time points was higher than those of the LipoIRI. This result indicated that the anti-EGFR antibody could help more efficiently transport LipoIRI to cells. 

The general pathway of nanoparticles internalized into cells is known to be phagocytosis, micropinocytosis, and caveolae-dependent and clathrin-mediated endocytosis. Herein, we used cytochalasin D as a phagocytosis and macropinocytosis inhibitor [20], amiloride as a macropinocytosis inhibitor [21], MBCD as an inhibitor of lipid rafts involved in caveolae-dependent endocytosis [22], and sucrose as a clathrin-mediated endocytosis inhibitor [23] to determine which pathway participates in cellular uptake. The results showed that the fluorescence intensities in cytochalasin D, amiloride, MBCD, and sucrose were significantly reduced, and the BS−LipoIRI group has a similar uptake level compared to LipoIRI. It indicated that the modified antibody does not change the uptake mechanism of nanoparticles. The upregulated cellular uptake efficiency was promoted by anti-EGFR specific targeting.

### 3.5. In Vitro Bxpc3 Cell Viability

**Figure 5:**
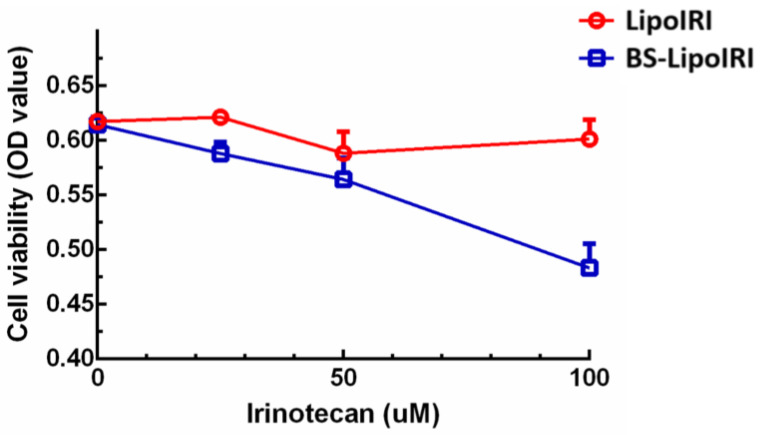
In vitro cytotoxicity of LipoIRI and BS−LipoIRI for BxPC3 pancreatic tumor cells.

BxPC3/EGFR pancreatic tumor cells were cultured with LipoIRI or BS−LipoIRI for 3 h to evaluate the cell viability by MTT assay. The results showed that a dose-dependent effect was observed with BS−LipoIRI on BxPC3 cells (Figure 5). The reason is probably that the anti-EGFR antibody promoted the transport of BS−LipoIRI into the cell, causing a significant difference in cell cytotoxicity.

### 3.6. PK Studies and Biodistribution Assessment of the BS−LipoIRI

**Figure 6:**
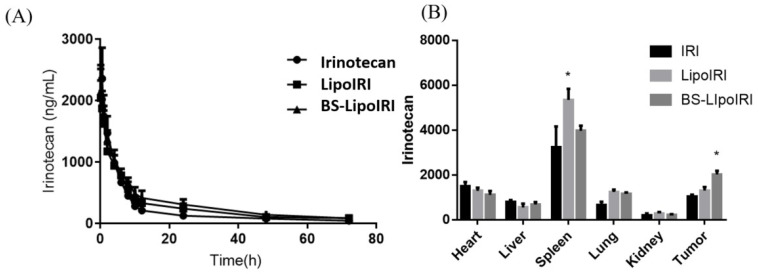
(**A**) Plasma concentration-time curves of irinotecan after intravenous administration of IRI, LipoIRI, and BS−LipoIRI at a dose of 20 mg/kg in rats. (**B**) Tissue distributions of irinotecan at 16 h after intravenous administration of IRI, LipoIRI, and BS−LipoIRI at a dose of 20 mg/kg into BxPC3 tumor-bearing SCID mice. * *p* < 0.01, a statistically significant difference compared with the IRI group.

The mean plasma concentrations of the drug after administration with a single dose of 10 mg/kg of irinotecan, LipoIRI, or BS−LipoIRI via jugular vein injection (with three Sprague Dawley rats per group) are shown in Figure 6. The PK profiles of LipoIRI and BS−LipoIRI were analyzed and compared with irinotecan. All PK profiles plotted in Figure 6A showed a high initial IRI concentration after the injection, followed by a rapid decline to the terminal phase, which gradually reached a steady-state concentration.

Biodistribution of irinotecan in major organs and tumor sites was assessed in BxPC-3 bearing mice at 8 h after IV injections of all treatments. As shown in Figure 6B, IRI accumulated in the spleen but showed a lower amount in the kidney. Interestingly, the statistically significantly higher IRI concentration was observed in the tumor site with BS−LipoIRI being 1.6-fold higher than the IRI group.

### 3.7. In Vivo Antitumor Efficacy in Tumor-Bearing Mice

**Figure 7:**
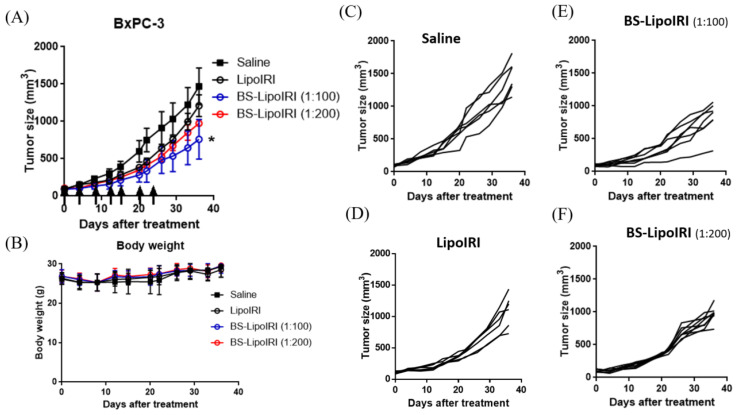
(**A**) The BxPC3 tumor growth curve after intravenous administration at a dosing regimen of 20 mg/kg IRI. (**B**) Body weight changes in tumor-bearing mice. Tumor size of individual mouse in the (**C**) Saline, (**D**) LipoIRI, (**E**) BS−LipoIRI (1:100), and (**F**) BS−LipoIRI (1:200) group. * *p* < 0.01, a statistically significant difference compared with the saline group.

To compare the in vivo therapeutic efficacy, the SCID mice bearing BxPC3 cells and WS1 cells were established as described above. After the tumor size reached approximately 150 mm^3^, the mice were treated with 20 mg/kg of irinotecan of different groups through the tail vein twice a week for 3 weeks. Tumor growth in BS−LipoIRI was greatly suppressed compared to those in saline or LipoIRI (Figure 7A). Furthermore, the BS−LipoIRI (1:100) group showed the greatest antitumor effect among all formulations in the BxPC3/WS1 cell-bearing mice. The percent of tumor volumes remaining after treatment with LipoIRI, BS−LipoIRI (1:200), and BS−LipoIRI (1:100) calculated with respect to that for the PBS treatment group (at 100%) were 72.3% ± 13.5%, 59.9% ± 10.9%, and 46.2% ± 12.8%, respectively. 

## 4. Discussion

Liposomes are thought to be a great drug carrier for cancer therapy, and there are many clinical products, such as Doxil^®^, Onivyde^®^, and Mepact^®^ [24,25,26]. The mechanism of liposomal drug delivery was indicated by previous studies, which revealed that the hydrophilic drugs are encapsulated into the central aqueous core of the liposome and are shielded from the body’s aqueous environment by the lipid bilayer [27,28]. After a while, the bilayer deteriorates, and the liposomes release their inner drug contents. Liposomal irinotecan has been developed for overcoming the pharmacological and clinical limitations of free irinotecan drugs [29,30]. It has a high-drug loading capacity and high stability in vivo, and was thought to enhance the drug permeability of the tumor site. However, the property of lacking active targeting ability causes most of the irinotecan drugs to accumulate in the body’s vital organs [31]. Although they have proven their benefit for clinical therapy, non-target particles still have side effects on the body or unstable therapeutic effects [32,33]. It is urgently needed to develop a tumor-specific targeting drug carrier. We first constructed a stable antitumor/anti-cancer-associated fibroblast dual-targeting BS−LipoIRI. Our results showed that BS−LipoIRI had a mean particle size of approximately 120 nm, drug encapsulation rate >75%, and drug loading approximately >7%, indicating its suitability for drug delivery to the tumor site in vivo by the EPR effect [34]. Moreover, the dual-targeting ability of BS−LipoIRI was revealed in the BxPC-3 (human pancreatic cancer cells) and WS-1 (human fibroblast cells) binding affinity experiments. In vivo biodistribution assay indicated that treatment with BS−LipoIRI would increase the drug level at the tumor site, and decrease the drug level in the spleen, which shows its specific targeting in the body [35].

In the previous studies, liposome has shown the property of slow drug release [36]. Our in-vitro drug release results indicated the drug release curve of the free drug, LipoIRI, and BS−LipoIRI did not have a significant difference. Recent research shows that cubosomes, the internal crystalline structure, provides a complex crystalline network for the entrapment and encapsulation of hydrophobic and hydrophilic molecules [37,38]. With the intrinsic slow disassembling of the crystalline structure in biological media, the cubosomes may provide a prolonged release of the transported drugs.

The cellular uptake of human pancreatic cancer cells showed that BS−LipoIRI could effectively internalize into cells and then LipoIRI. This result might be due to the specific targeting of antibodies. To examine the cellular uptake pathway of cancer cells, we used different inhibitors to block phagocytosis, micropinocytosis, and caveolae-dependent or clathrin-mediated endocytosis [39,40,41]. Every inhibitor could block the particle cellular uptake efficacy, regardless of the liposomes constructed with the antibody, indicating that all uptake pathways were involved. Notably, the group of sucrose inhibitors for BS−LipoIRI showed considerably lower mean fluorescence intensities than any other group, indicating that sucrose, as a clathrin-mediated endocytosis inhibitor, could seriously affect the BS−LipoIRI cellular uptake. The further mechanisms between bispecific antibody- and clathrin-mediated endocytosis could be studied in the future. 

The in vivo plasma pharmaceutic kinetic results showed that LipoIRI or BS−LipoIRI maintained slightly higher drug concentration than irinotecan. The result is similar with that of previous studies that liposome formulation could prolong drug concentration in the circulation, which is beneficial for further therapeutic effect [30,42]. Moreover, the biodistribution results indicated that the BS−LipoIRI group had higher irinotecan drug concentration at the tumor site, and the antibody-targeting liposome could delivery drug from the circulation to the tumor. The in vivo human pancreatic cancer xenograft mouse model confirmed that BS−LipoIRI could effectively inhibit tumor growth, which could be attributed to the increasing drug concentration in the tumor. Overall, BS−LipoIRI showed the specific targeting ability and great suppression against pancreatic tumor.

## 5. Conclusions

In this study, we successfully constructed an anti-EGFR/anti-FAP bispecific targeting antibody on irinotecan liposomes. The results showed that BS−LipoIRI has a great targeting effect on human pancreatic cancer cells and fibroblasts. The in vivo biodistribution experiments indicated that BS−LipoIRI (1:100) accumulated in the tumor site, with BS−LipoIRI being 1.6-fold higher than the IRI group, thus generating the greatest antitumor effect on the human pancreatic tumor-bearing mouse model, which decrease about 46.2% of tumor volume compared with the saline group. Overall, BS−LipoIRI showed its dual-targeting property for cancer treatment and could be a promising cancer drug carrier for further clinical application. 

## Figures and Tables

**Table 1:** Stability of BS−LipoIRI (1:100) after 2, 4, and 6 weeks of storage. The average particle size, PDI, and zeta potential are expressed as mean ± SE of three independent experiments.

Time	Z-Ave (nm)	PDI	Zeta Potential (mv)	Drug Loading (%)	Drug Encapsulation (%)
**2 Weeks**	114.6 ± 0.16	0.257 ± 0.0255	−7.21 ± 0.181	8.23%	78.11%
**4 Weeks**	116.8 ± 0.31	0.246 ± 0.1284	−8.11 ± 0.523	8.10%	77.93%
**6 Weeks**	115.7 ± 0.18	0.592 ± 0.1119	−7.33 ± 0.115	7.43%	71.27%
